# Use of axonal projection patterns for the homologisation of cerebral nerves in Opisthobranchia, Mollusca and Gastropoda

**DOI:** 10.1186/1742-9994-10-20

**Published:** 2013-04-18

**Authors:** Annette Klussmann-Kolb, Roger P Croll, Sid Staubach

**Affiliations:** 1 Biosciences, Institute of Ecology, Evolution and Diversity, Phylogeny and Systematics group, Goethe University, Max-von-Laue-Straße 13, Frankfurt am Main, 60438, Germany; 2 Department of Physiology and Biophysics, Dalhousie University, Halifax, Canada

**Keywords:** Opisthobranchia, Cephalic sensory organs, CSO, Cerebral nerves, Axonal tracing, Homology

## Abstract

**Introduction:**

Gastropoda are guided by several sensory organs in the head region, referred to as cephalic sensory organs (CSOs). These CSOs are innervated by distinct nerves. This study proposes a unified terminology for the cerebral nerves and the categories of CSOs and then investigates the neuroanatomy and cellular innervation patterns of these cerebral nerves, in order to homologise them. The homologisation of the cerebral nerves in conjunction with other data, e.g. ontogenetic development or functional morphology, may then provide insights into the homology of the CSOs themselves.

**Results:**

Nickel-lysine axonal tracing (“backfilling”) was used to stain the somata projecting into specific nerves in representatives of opisthobranch Gastropoda. Tracing patterns revealed the occurrence, size and relative position of somata and their axons and enabled these somata to be mapped to specific cell clusters. Assignment of cells to clusters followed a conservative approach based primarily on relative location of the cells. Each of the four investigated cerebral nerves could be uniquely identified due to a characteristic set of soma clusters projecting into the respective nerves via their axonal pathways.

**Conclusions:**

As the described tracing patterns are highly conserved morphological characters, they can be used to homologise nerves within the investigated group of gastropods. The combination of adequate number of replicates and a comparative approach allows us to provide preliminary hypotheses on homologies for the cerebral nerves. Based on the hypotheses regarding cerebral nerve homology together with further data on ultrastructure and immunohistochemistry of CSOs published elsewhere, we can propose preliminary hypotheses regarding homology for the CSOs of the Opisthobranchia themselves.

## Background

The Opisthobranchia comprise a specialised and highly evolved group of mostly marine slugs and snails within the Heterobranchia with up to 6000 extant species confined to nine taxa [1]. Recent studies on phylogenetic relationships within Heterobranchia suggest a paraphyly of Opisthobranchia, with some taxa showing closer relationships to traditional pulmonate taxa than to other Opisthobranchia [2-4]. The cephalic sensory organs (CSOs) provide a prominent but variable character complex in these taxa, with each subgroup possessing a more-or-less characteristic set of CSOs [5]. Within the Acteonoidea, Cephalaspidea and some Acochlidiacea, the CSOs manifest as the lip organ, Hancock’s organ and cephalic shield [6-12]. The taxon Nudipleura is divided into the Nudibranchia, which show a variety of labial tentacles, oral veils, solid rhinophores and Hancock’s organ, and the Pleurobranchomorpha, which possess prominent oral veils and curled rhinophores [8,13]. In some Aplysiomorpha oral lobes and a Hancock’s organ are present, but in general most Aplysiomorpha only bear labial tentacles and rhinophores [14].

Homology of the different types of CSOs in the various opisthobranch subgroups has not yet been studied in detail, although earlier investigations proposed hypotheses of homology of CSOs within Opisthobranchia based primarily on their patterns of innervations by presumably homologous nerves [7,8,13,15-17]. However, the nerves themselves were primarily homologized with respect to their ganglionic origin and their peripheral terminations, i.e., which CSO they innervated. Moreover, the high variability of nervous innervation patterns of homologous structures found in Crustacea [18] and other invertebrates [19-21] suggests a further need to refine this criterion for assessment of homology for the cerebral nerves and in consequence the CSOs.

In the present paper we propose a novel complex character trait for the evaluation of homology of the cerebral nerves by using an axonal filling/tracing technique (“backfilling”, i. e. filling the nerve from the distal end). This results in the staining of individual somata projecting into each nerve. These somata can be grouped into clusters by their close location and by their common axonal projection patterns. Moreover, backfilling of the somata allows the visualization of complex details in their morphologies [22]. Homology at the cellular level has already been discussed in Gastropoda [23-27] and in Crustacea [21] and several criteria for cellular homology have been established. Additionally, our own previous studies led to the proposition of extended criteria for homology of axonal tracing patterns [10,11]. These criteria comprise:

1) the number of cerebral cell clusters projecting into the respective nerve; each cluster represents cells or regions with particular projections;

2) the position of the cell clusters in relation to each other and to ganglionic structures, like nerve roots, commissures and connectives;

3) the distribution of axonal pathways; whereas the final arborization of the axons can be highly variable [21,24], the general pathways of major bundles of axons seem to be relatively invariant [10,11].

The current study serves as an extended test of whether patterns of individual neurons or clusters of somata and their respective axons can be used as a morphological character complex for the homologization of nerves.

A re-evaluation of the cerebral nerves is particularly needed, because there is much confusion about terminology and even the characteristic number of the cerebral nerves in Opisthobranchia [7,8,15]. Thus we propose a unified terminology for cerebral nerves (following [7]) and the categories of CSOs. In the current study we examine the innervation patterns and cellular origins of the four cerebral nerves which innervate the CSOs in three opisthobranch taxa. We focus on the tracing patterns of *Aplysia punctata* and *Aplysia californica* (Aplysiomorpha), *Archidoris pseudoargus* (Nudibranchia) and *Pleurobranchaea meckeli* (Pleurobranchomorpha) possessing different sets of CSOs. We compare these cellular innervations patterns with earlier investigations [10,11] of *Acteon tornatilis* (Acteonoidea) and *Haminoea japonica* (Cephalaspidea) (in the former studies erroneously identified as *Haminoea hydatis*) to further test interspecific variability. Our study aims at examining whether variability in morphology of CSOs is reflected by differences in the patterns of somata projecting into the nerves innervating the organs. The earlier investigations in *H. japonica* and *A. tornatilis* showed little variation between the axonal tracing patterns. Both species share similar but not equal sets of CSOs, which have been assumed to be an adaptation to their life history [10,11]. Therefore, similarity of the axonal tracing patterns could be explained by sharing a similar gross morphology or a similar functional adaptation of the CSOs. The investigation and comparison of further opisthobranch species in this study clarifies the interspecific variability of axonal tracing patterns at a higher taxonomic, morphological and functional level. Finally, we postulate preliminary hypotheses of homology for the CSOs based on the homology of the cerebral nerves innervating them. Nevertheless, innervation by homologous nerves does not necessarily reflect homology of the organs they innervate [28]. Due to the extreme diversity of the CSOs within the Opisthobranchia, parallelism cannot be excluded.

## Results

### Nerves innervating CSOs

Previous terminology for the nerves innervating various organs or structures in Gastropoda has often been inconsistent and confusing, thus hampering comparisons of nervous innervation patterns across taxa. Within the investigated Opisthobranchia we found a basic pattern of four cerebral nerves, which has also been described for the Opisthobranchia by Huber [8]. However, there is no common notation of these nerves in earlier investigations, e.g. [8,15,16,29-31]. Table 1 summarizes terms used for the four cerebral nerves in Opisthobranchia, in representative studies on the neuroanatomy of these gastropods. The most common synonyms for the cerebral nerves and their innervation area are shown. In the present study, we used a modified terminology from Edlinger [7] who focussed his investigations of neuroanatomy of the CSOs on Acteonoidea and Cephalaspidea. However, instead of using Latin names, numbers were used as notations of the cerebral nerves, as it was also done by Vayssière [29].

**Table 1:** Terminology of cerebral nerves in Opisthobranchia

**Modified synonyms after Edlinger**[7] used in the present study	**Vayssière**[29]	**Hanström**[15]	**Huber**[8]	**Croll et al.**[31]	**Innervated CSO/Head region** See also Edlinger [7], Huber [8]
N1	c1	Nervus labialis minor, Nervus oralis	Nervus labialis superior, Nervus oralis	Upper labial nerve	Lip
N2	c3	Nervus labialis superior, Nervus tentacularis	Nervus labialis, Nervus labiotentacularis, Nervus menti	anterior tentacle nerve	ASOs: Anterior tentacle, Lip organ, oral veil, oral lobe, anterior Hancock’s organ
N3	c4	Nervus tentacularis, Nervus rhinophoralis	Nervus rhinophoralis	posterior tentacle nerve	PSOs: Rhinophore, posterior Hancock’s organ, posterior tentacle
Nclc	c2	Nervus proboscidis	Nervus tentacularis, Nervus clypei-capitis	lower labial nerve	Anterior / lateral body wall, cephalic shield, cephalic disc

**Figure 1:**
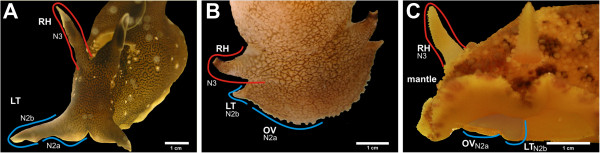
**Cephalic sensory organs (CSOs) within the Opisthobranchia. A**: *Aplysia punctata*, picture showing the rhinophores and the labial tentacles. **B**: *Pleurobranchaea meckeli*, picture showing the rhinophores, the labial tentacles and the oral veil. **C**: *Archidoris pseudoargus,* picture showing the rhinophores, the labial tentacles and the oral veil, usually the labial tentacles and the oral veil are completely covered by the massive mantle. In the picture shown, part of the mantle is upturned and the labial tentacles and the oral veil are highlighted. Abbreviations (referring to all subfigures, colors indicate the nervous innervation of the CSOs). N2 (N2a= inner branch, N2b= outer branch) in blue and the N3 in red, LT – labial tentacle, OV – oral veil, RH – rhinophore.

**Figure 2:**
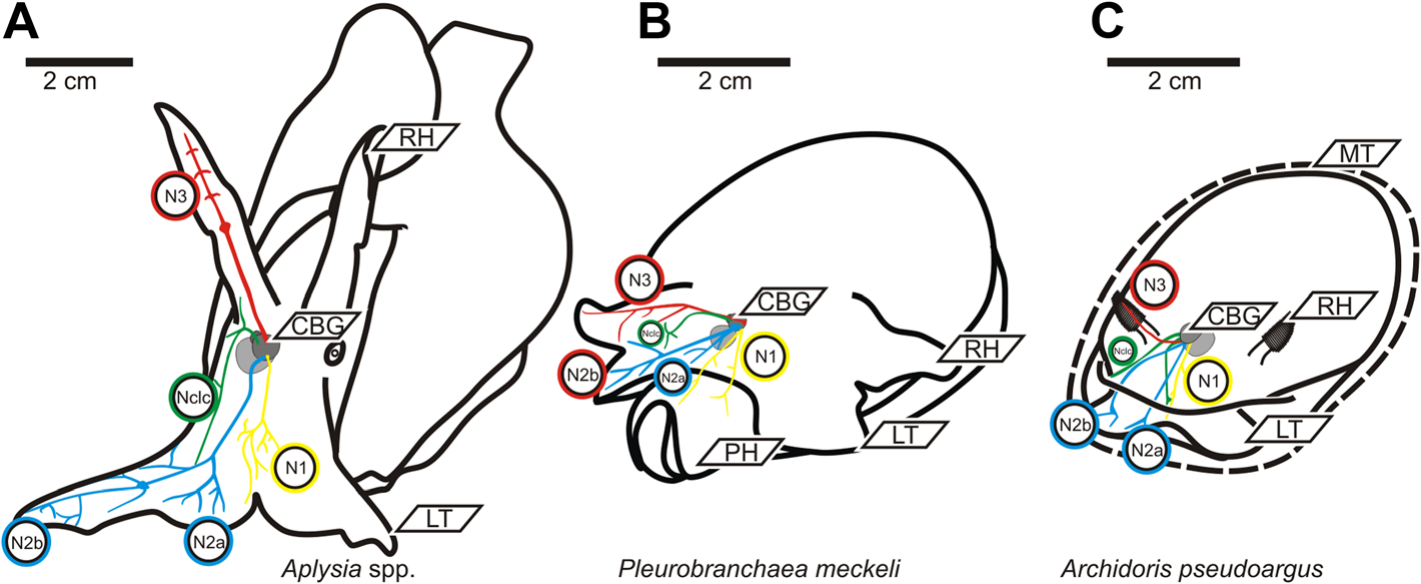
**Neuroanatomy. A**: Neuroanatomical scheme of the four cerebral nerves of the investigated Aplysiomorpha, the two investigated *Aplysia* show no significant differences (only right hemisphere shown). **B**: Neuroanatomical scheme of the four cerebral nerves of the investigated Pleurobranchomorpha *Pleurobranchaea meckeli* (only right hemisphere shown). **C**: Neuroanatomical scheme of the four cerebral nerves of *Archidoris pseudoargus* (only right hemisphere shown). Abbreviations (referring to all subfigures): CBG – cerebral ganglia, LT – labial tentacle, N1 in yellow, N2 (N2a= inner branch, N2b= outer branch) in blue, N3 in red and the Nclc in green; OV – oral veil, RH – rhinophore.

The cerebral nerves, investigated by macroscopic dissection, were thus numbered from anterior to posterior, as follows. The *nervus oralis*, which innervates the most anterior CSO, namely the lip, is termed as the N1. The *nervus labialis*, which is divided in two branches within the Opisthobranchia and innervates the other anterior CSOs is named N2. In this study we use the generalized term anterior sensory organ (ASO) for these CSOs. As the N2 is bifurcated and the branches innervate different organs, we furthermore separate the ASOs into ASOa (innervated by the inner branch of the N2) and ASOb (innervated by the outer branch of the N2). The *nervus rhinophoralis* which provides the posterior CSOs (PSOs) is termed N3. The fourth cerebral nerve, the *nervus clypei capitis* innervates parts of the body wall or the cephalic shield but is not primarily correlated to a sensory organ. Therefore this nerve has not been numbered and we term this nerve as Nclc. A summary of the innervated area by each nerve for each species and their respective category is shown in Table 2. Additionally some of the CSOs of the investigated species are shown exemplarily in Figure 1 with an indication of their nervous innervation which is then shown in detail and schematically in Figure 2.

**Table 2:** Innervation of CSOs by cerebral nerves; categories of CSOs

**Nerve**	***Aplysia punctata***	***Archidoris pseudoargus***	***Pleurobranchea meckeli***	***Acteon tornatilis***[10]	***Haminoea japonica***[11]	**CSO categories**
N1	lip	lip	lip	lip	lip / anterior cephalic shield	no category
N2 inner branch	basal part of the labial tentacle	inner part of the oral tentacle/oral lobe	oral veil	anterior groove among the anterior cephalic shield	lip organ	ASOa
N2 outer branch	tip of the labial tentacle	outer part of the oral tentacle/oral lobe	labial tentacle	posterior groove among the anterior cephalic shield	anterior Hancocks organ	ASOb
N3	rhinophore	rhinophore	rhinophore	possibly reduced in *Acteon*	posterior Hancocks organ	PSO
Nclc	anterior/lateral body wall	anterior/lateral body wall	anterior/lateral body wall	posterior cephalic shield	posterior cephalic shield	no category

In our studies, we observed only very few morphological differences in cerebral nerve anatomy and tracing patterns between the two Aplysiomorpha, *Aplysia californica* and *Aplysia punctata* (Figure 2A). Therefore, both species will be treated as *Aplysia* spp.

In general, we found that the N1 innervates the lip and anterior head region. The N2 innervates the labial tentacles, and the N3 innervates the rhinophores, while the Nclc innervates the anterior and posterior body wall, and also the lower part of the labial tentacles. In both species of *Aplysia*, the inner branch of the N2 (N2a) innervates the thick base of the labial tentacles whereas the outer branch N2b provides the outer lobe of the labial tentacles.

In the investigated Pleurobranchomorpha, *Pleurobranchaea meckeli*, N1 innervates the lip (Figure 2B). The bifurcated N2 innervates the oral veil, labial tentacle and groove with the inner branch (N2a) innervating the median part of the oral veil, and the outer branch (N2b) innervating the labial tentacles. The N3 innervates the rolled rhinophores and forms a small rhinophoral ganglion directly above the cerebral ganglion. The Nclc innervates parts of the anterior and posterior body wall.

The investigated Nudibranchia, *Archidoris pseudoargus* (Figure 2C), also shows a very similar distribution of the cerebral nerves as found in the Pleurobranchomorpha species described above. The N1 innervates the lip, the bifurcated N2 the labial tentacles, the N3 innervates the massive rhinophores which possess disc shaped structures at the distal part, with also a rhinophoral ganglion at the base of the nerve and the Nclc innervates the lateral and anterior head region.

### Axonal tracing patterns

**Figure 3:**
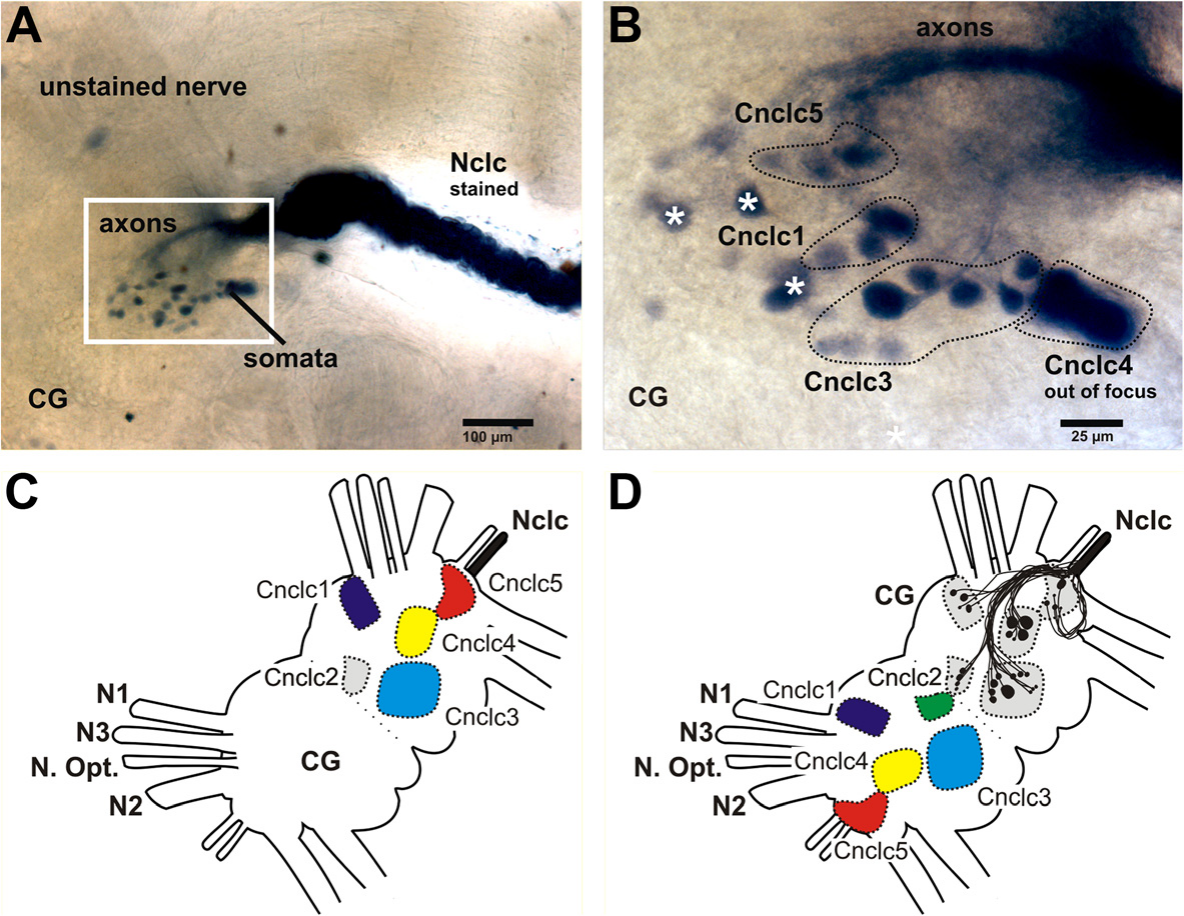
**Scheme for the development of the axonal tracing patterns as shown in Figures**4**,**5**,**6, The first image (**A**) shows a photograph of part of the right hemisphere of the cerebral ganglion for an axonal tracing of the Nclc of *Aplysia punctata*. The stained nerve, axons and somata are labeled. **B** shows a close-up of A and several clusters are indicated. The white asterisks mark somata which are not found in all replicates and the cluster Cnclc2 (see C and D) is not visible here. The schematic in Figure **C** shows the first step in developing the schematics shown in Figures 4, 5, 6. First the location of the clusters is determined by axonal tracings in several replicates viewed from different angles. After the common position is defined, the clusters are color-coded, and finally in **D** mirrored to the other hemisphere and the common axonal pathways and somata are added in the original hemisphere. It is important to understand that **D** is a schematic based on multiple replicates (including **B**) and that somata which have not been found in all replicates are not shown in **D**.

**Figure 4:**
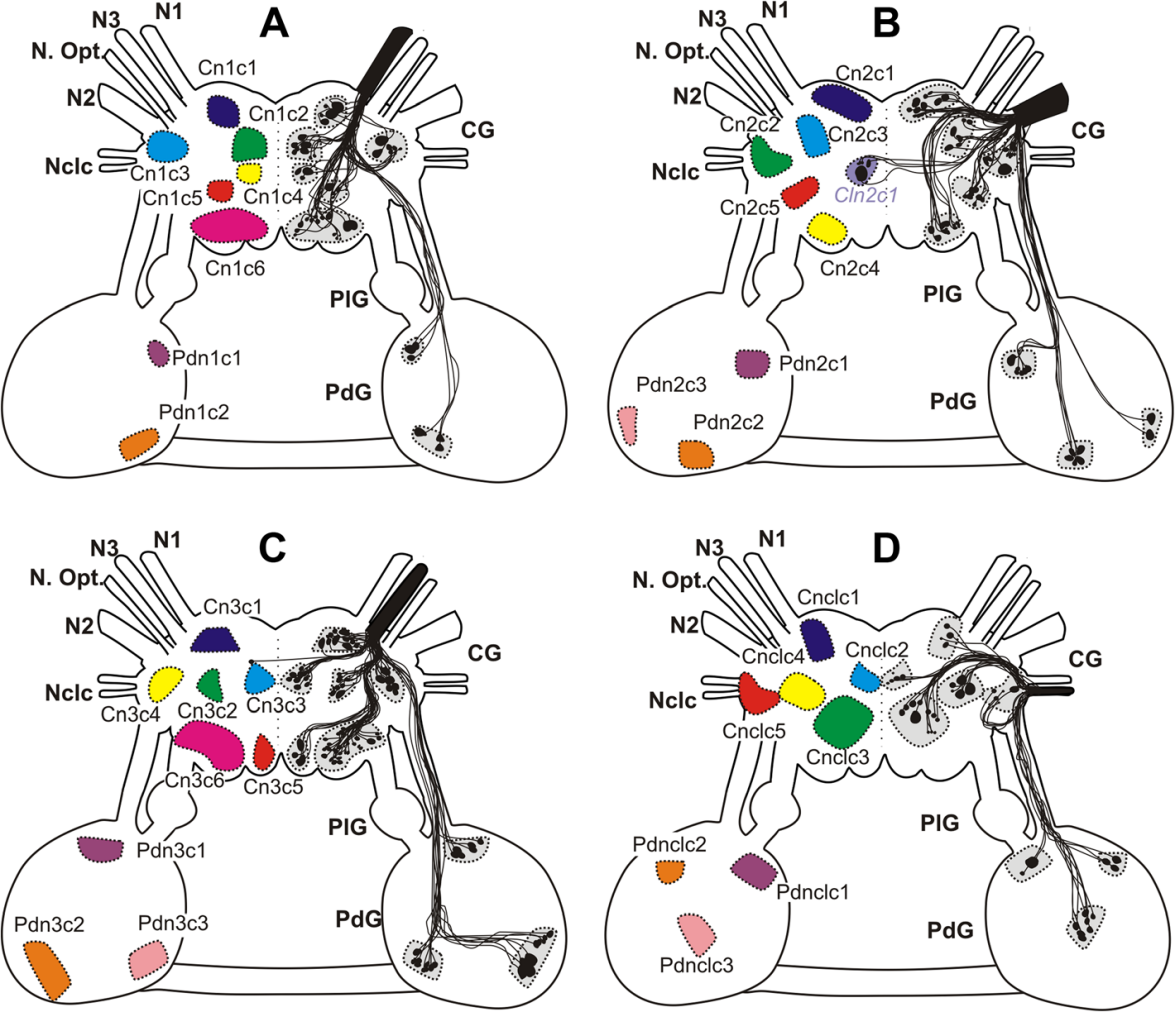
**Cellular innervation patterns of the cerebral nerves in *Aplysia spp. ***Schematic outline of somata and their axons projecting into the N1 (**A**), N2 (**B**), N3 (**C**) and Nclc (**D**). The size and position of the somata are digitalized from camera lucida drawings, the distribution of the axons are averaged over all replicates. CG - cerebral ganglia, N1 - Nervus oralis, N2 - Nervus labialis, N3 - Nervus rhinophoralis, Nclc - Nervus clypei capitis, N. Opt. - Nervus opticus, , PdG - pedal ganglia, PlG - pleural ganglia.

**Figure 5:**
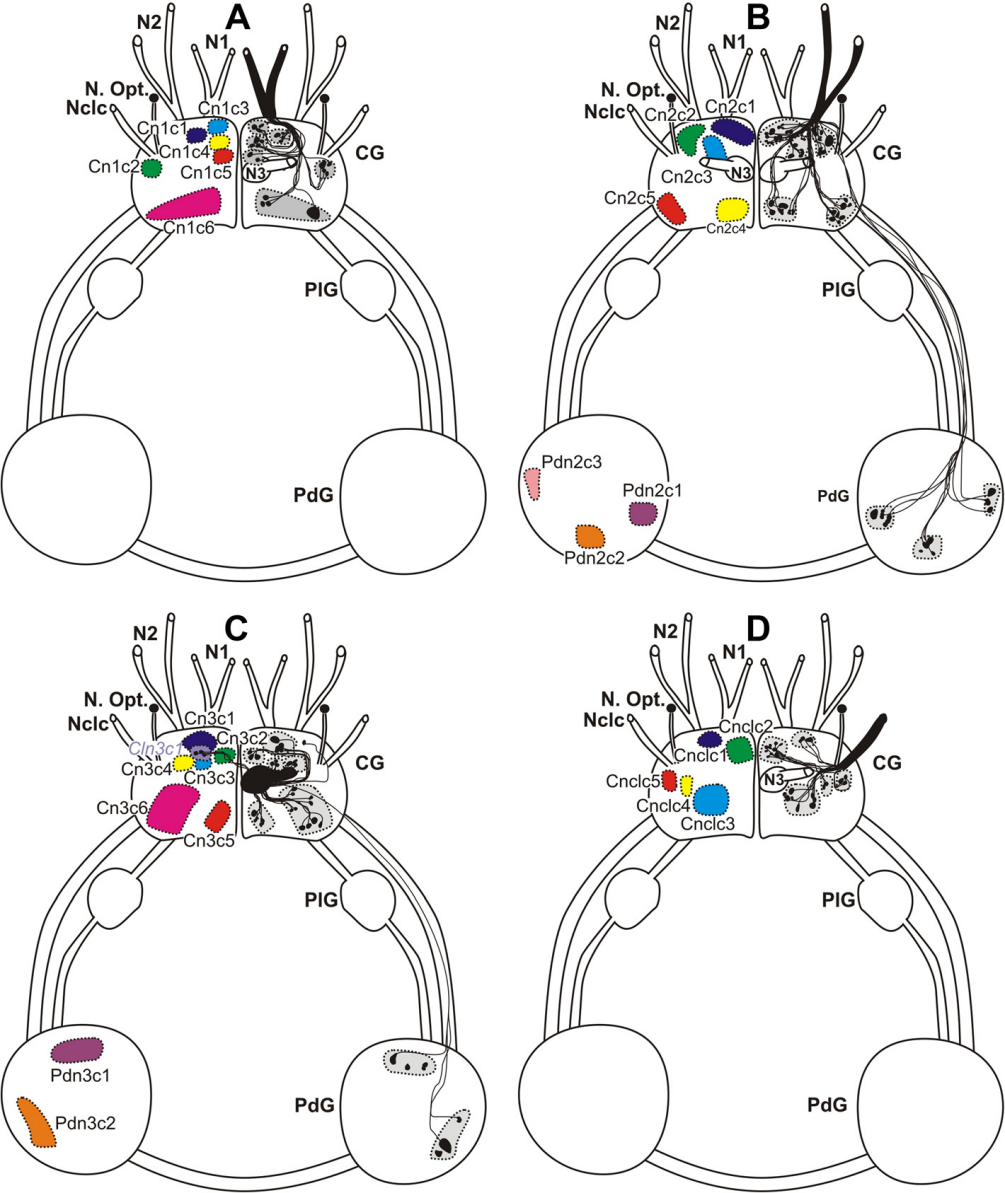
**Cellular innervation patterns of the cerebral nerves in *Pleurobranchaea meckeli. ***Schematic outline of somata and their axons projecting into the N1 (**A**), N2 (**B**), N3 (**C**) and Nclc (**D**). The size and position of the somata are digitalized from camera lucida drawings, the distribution of the axons are averaged over all replicates. CG - cerebral ganglia, N1 - Nervus oralis, N2 - Nervus labialis, N3 - Nervus rhinophoralis, Nclc - Nervus clypei capitis, N. Opt. - Nervus opticus, PdG - pedal ganglia, PlG - pleural ganglia.

**Figure 6:**
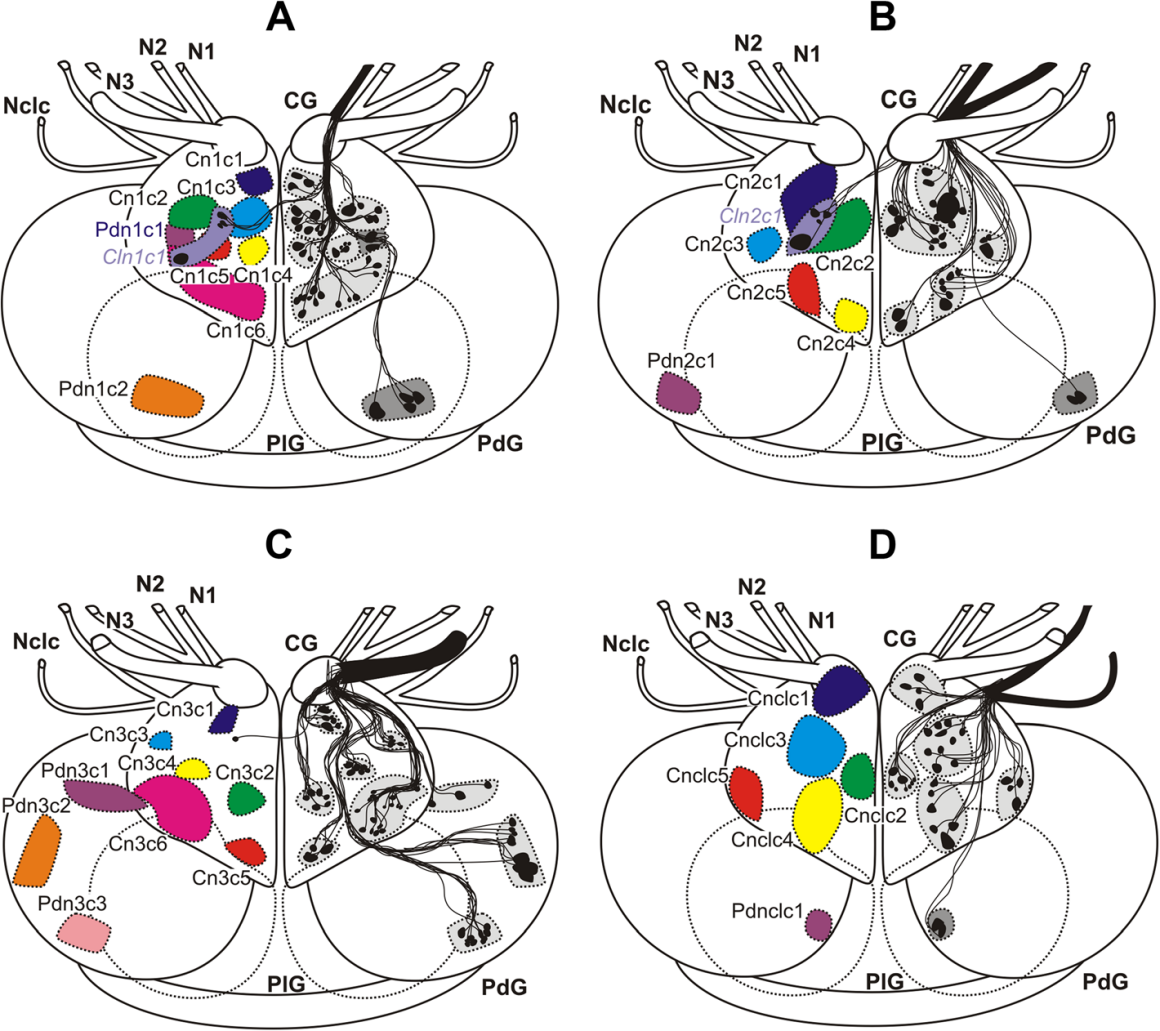
**Cellular innervation patterns of the cerebral nerves in *Archidoris pseudoargus. ***Schematic outline of somata and their axons projecting into the N1 (**A**), N2 (**B**), N3 (**C**) and Nclc (**D**). The size and position of the somata are digitalized from camera lucida drawings, the distribution of the axons are averaged over all replicates. CG - cerebral ganglia, N1 - Nervus oralis, N2 - Nervus labialis, N3 - Nervus rhinophoralis, Nclc - Nervus clypei capitis, PdG - pedal ganglia, PlG - pleural ganglia.

Figure 3 shows our approach to develop schematic representations of cell clusters and somata recognized by axonal tracing based on multiple replicates. As in our previous study [11], we defined clusters of nerve cells here on the basis of their close proximity within the ganglia and the tight fasciculation of their axons projecting into the filled nerve. Assignment of cells to clusters followed a conservative approach based primarily on relative location of the cells. Some clusters are clearly recognizable by spatial separation, in other cases the clusters were defined by the proximity of axonal pathways through the different planes of the sample (not visible in photographs). The schematic figures (Figures 4, 5, 6), which represent idealized schematics of all investigated replicates, only show the minimum number of somata found in each replicate, therefore cells only found in single replicates are not represented in the schematics. In general, the tracing patterns for the cerebral and pedal clusters identified showed distinct separations and were easy to identify. The control samples for the left hemisphere of the CNS showed no significant differences. Table 3 shows the minimum and maximum number of somata found in all replicates, e.g. 5 + 2, for each traced nerve and each species.

**Table 3:** Number of somata found in each cluster within the investigated species; The identified clusters were named with abbreviations signifying the ganglion in which they are located, the nerve filled and a number indicating the order of their description (for example, Cn1c3: **C**erebral **N1 c**luster **3**; Pdn1c2: **P**e**d**al **N1 c**luster **2**)

**Clustername**	***Aplysia *spp.**	***P. meckeli***	***A. pseudoargus***	**Clustername**	***Aplysia *spp.**	***P. meckeli***	***A. pseudoargus***
**N1**				**N3**			
Cn1c1	5+2	4+1	3+2	Cn3c1	12+3	5+2	6+1
Cn1c2	8+2	4+2	7+2	Cn3c2	10+3	3+1	6+2
Cn2c3	7+1	7+2	3+1	Cnclc3	11+3	8+4	9+3
Cn1c4	3+1	4+3	3+3	Cn3c4	6+1	3+2	6+2
Cn1c5	6+3	2+0	5+2	Cn3c5	4+1	6+4	4+2
Cn1c6	16+4	4+2	14+5	Cn3c6	28+6	10+2	18+4
*Cln1c1*	/	/	4+1	*Cln3c1*	/	3+0	/
Pdn1c1	3+1	/	2+0	Pdn3c1	6+1	4+1	3+0
Pdn1c2	3+3	/	4+1	Pdn3c2	9+2	5+2	9+3
				Pdn3c3	10+2	/	10+3
**∑ cerebral**	**48**	**48**	**28**	**∑ cerebral**	**30**	**26**	**29**
**∑ pedal**	**13**	**14**	**2**	**∑ pedal**	**12**	**0**	**2**
**Clustername**	***Aplysia *spp.**	***P. meckeli***	***A. pseudoargus***	**Clustername**	***Aplysia *spp.**	***P. meckeli***	***A. pseudoargus***
**N2**				**Nclc**			
Cn2c1	11+2	13+2	8+1	Cnclc1	4+2	3+0	4+1
Cn2c2	12+3	10+1	9+2	Cnclc2	5+1	10+2	8+3
Cn2c3	7+1	7+2	3+1	Cnclc3	11+3	8+4	9+3
Cn2c4	10+2	10+1	6+2	Cnclc4	5+2	2+1	7+1
Cn2c5	8+3	8+2	2+0	Cnclc5	5+2	3+0	5+2
*Cln1c1*	5+0	/	4+0	Pdnclc1	2+2	/	2+3
Pdn2c1	6+1	3+2	2+1	Pdnclc2	3+2	/	/
Pdn2c2	5+2	4+2	/	Pdnclc3	7+4	/	/
Pdn2c3	2+0	7+3	/				
**∑ cerebral**	**48**	**48**	**28**	**∑ cerebral**	**30**	**26**	**29**
**∑ pedal**	**13**	**14**	**2**	**∑ pedal**	**12**	**0**	**2**

We based our modified terminology for the comparison of the axonal tracing patterns upon that proposed by Staubach et al. [11] for the cephalaspidean *Haminoea japonica*.

### *Axonal tracing patterns of aplysia* spp

The axonal tracing patterns of the two aplysiomorph species showed no significant differences. Typical axonal tracing patterns for each of the four cerebral nerves of *Aplysia* spp. are shown in Figure 4 and the number of somata in each of the clusters in Table 3. For the N1 (n = 20) we were able to characterize six cerebral clusters (Cn1c1-6; C = cerebral) in each sample (Figure 4A). The maximum number of somata (= up to 20) was found in Cn1c6 (Table 3). Additionally we found two pedal clusters (Pdn1c1-2). The cerebral clusters were distributed over the whole cerebral ganglion. The tracing pattern of the N2 (n = 20) consisted of five cerebral clusters (Cn2c1-5), a contralateral cerebral cluster (*Cln2c1*; Cl = contralateral) and three pedal clusters (Pdn2c1-3; Pd = pedal) (Figure 4B). The third traced cerebral nerve (n = 20) was the N3. Six cerebral clusters (Cn3c1-6), one contralateral soma and three pedal clusters (Pdn3c1-3) were identified (Figure 4C). In the Nclc, the axonal tracing pattern (n = 10) consisted of five cerebral clusters (Cnclc1-5) (Figure 4D) and three pedal clusters (Pdnclc1-3).

### Axonal tracing patterns of pleurobranchaea meckeli

The characteristic axonal tracing patterns of labelled somata for all four cerebral nerves of *P. meckeli* are shown in Figure 5A-D and Table 3, including the approximate pathways of the stained axons. For the N1 (n = 8) we were able to identify six cerebral clusters (Cn1c1-6) in each sample (Figure 5A). The cerebral clusters were primarily distributed in the median anterior region of the cerebral ganglion, except for Cn1c2 and Cn1c6. The tracing pattern of the N2 (n = 9) consisted of five cerebral clusters (Cn2c1-5) and three pedal clusters (Pdn2c1-3) (Figure 5B). The third traced cerebral nerve was the N3 (n = 10). Six cerebral (Cn3c1-6), one contralateral cerebral (*Cln3c1*) and two pedal clusters (Pdn3c1-2) were identified (Figure 5C). The contralateral cluster was located in the anterior region of the cerebral ganglion near the base of the N2. In the Nclc (n = 10), the tracing pattern consisted of five cerebral clusters (Cnclc1-5) (Figure 5D). Unlike in the other investigated species we found no pedal clusters for the N1 and the nclc.

### Axonal tracing patterns of archidoris pseudoargus

The characteristic axonal tracing patterns of all four cerebral nerves of *A. pseudoargus* are shown in Figure 6A-D and Table 3. As in the other examined species, six cerebral clusters (Cn1c1-6) were found backfilling the N1 (Figure 6A). Additionally, we found a cerebral contralateral cluster (*Cln1c1*). Moreover, two pedal clusters (Pdn1c1-2) were present (Figure 6A). Again the variation between the samples was restricted to very few somata in some clusters. The cerebral clusters were distributed across the entire cerebral ganglion. Due to the fusion of the ganglia in *A. pseudoargus*, the clusters were not well separated, also the pedal cluster Pdn1c1 (abbreviation in blue, Figure 6A) was located directly underneath the cerebral clusters. The tracing pattern of the N2 (n = 10) consisted of five cerebral clusters (Cn2c1-5), a contralateral cerebral cluster (*Cln2c1*) and a pedal cluster (Pdnlc1) (Figure 6B). Here the cerebral clusters showed distinct spatial separations and were also easy to identify, like in *Aplysia* spp*.* and *P. meckeli*. The third traced cerebral nerve (n = 10) was the N3. Six cerebral (Cn3c1-6), one contralateral soma and three pedal cluster (Pdn3c1-3) were identified (Figure 6C). The contralateral soma was located near the base of the N3 like the contralateral cluster *Clnrc1* in *P. meckeli*. Again we only observed slight intraspecific variability between the samples. In the Nclc (n = 10), the tracing pattern consisted of five cerebral clusters (Cnclc1-5) (Figure 6D) and one pedal cluster (Pdnclc1).

## Correlation analyses

The results of the Pearson Correlation Analyses, using the absolute numbers of somata within the cerebral clusters from Table 3 are shown in Table 4. Pedal and contralateral clusters were not analysed due to the incongruence of their presence within the investigated species.

**Table 4:** Pearson correlation analyses for the number of somata in the cerebral cluster

**N1 cerebral**	***Aplysia *spp.**	***P. meckeli***	***A. pseudoargus***
***Aplysia *spp.**	/		
***P. meckeli***	0.185	/	
***A. pseudoargus***	0.965	0.836	/
**N2 cerebral**	***Aplysia *spp.**	***P. meckeli***	***A. pseudoargus***
***Aplysia *spp.**	/		
***P. meckeli***	0.795	/	
***A. pseudoargus***	0.956	0.79	/
**N3 cerebral**	***Aplysia *spp.**	***P. meckeli***	***A. pseudoargus***
***Aplysia *spp.**	/		
***P. meckeli***	0.795	/	
***A. pseudoargus***	0.965	0.836	/
**Nclc cerebral**	***Aplysia *spp.**	***P. meckeli***	***A. pseudoargus***
***Aplysia *spp.**	/		
***P. meckeli***	0.471	/	
***A. pseudoargus***	0.724	0.723	/

The correlation analyses for the cerebral clusters of the N1 nerve show the lowest but also the highest correlation values within all analyses, with a high correlation coefficient of 0.967 between *Aplysia* spp. and *A. pseudoargus* but a very low correlation coefficient of 0.115 between *A. pseudoargus* and *P. meckeli* and 0.185 between *Aplysia* spp. and *P. meckeli*. The correlation coefficients for the N2 rank between 0.79 and 0.965, N3 between 0.795 and 0.965 and Nclc between 0.471 and 0.724.

In detail we found the highest correlation between the number of somata within the cerebral clusters of *Aplysia* spp. and *A. pseudoargus* for all nerves. Within the N1 the correlation is lowest between *P. meckeli* and *A. pseudoargus*. We found the same state in the analyses for the N2. In the analyses of the N3 and Nclc cerebral somata number we found higher coefficients for *P. meckeli* with *A. pseudoargus* than within the analyses for *Aplysia* spp. and *P. meckeli*.

## Discussion

The major aim of this study was to test whether patterns of backfilled neurons can be used as a morphological character complex for the homologisation of cerebral nerves in opisthobranch gastropods. Here we tested whether the criteria for evaluation of homology of the axonal tracing patterns, based on the earlier intra- and interspecific studies [10,11] can be confirmed by extending the comparison of interspecific innervation patterns. Furthermore we wanted to homologise the cerebral nerves and in turn to postulate primary hypotheses of homology for the CSOs.

### Intra- and interspecific variability of tracing patterns

Earlier investigations on *Haminoea japonica*[11] have shown that the number and the size of the somata within a distinct cluster are correlated with the body size of the individual, whereas the number of clusters projecting into a particular nerve was constant within a species. *H. japonica* and *Acteon tornatilis* are relatively small species, which reach sexual maturity when body length approaches 1,5 cm but individuals can reach a body length up to 3 cm (own investigations, unpublished data), *Pleurobranchaea meckeli* and *Archidoris pseudoargus* are medium sized (up to 10 cm body length) and *Aplysia* spp. are large species (up to 30 cm). Besides the body size *P*. *meckeli* has the largest CNS due to elongated commissures and connectives rather than ganglion size, whereas the CNS of *Aplysia* spp. and *A. pseudoargus* are nearly the same size, regardless of the body length over the range of animal sizes examined.

Throughout our investigation of several taxa of opisthobranch gastropods in the frame of this study and in previous investigations, we could identify uniform tracing patterns via four cerebral nerves which can be attributed to characteristic neuronal cell clusters in the CNS. All investigated opisthobranchs have four major cerebral nerves which innervate the head region (optic nerve excluded). We found that the cellular innervation patterns for the different nerves in the different taxa were highly conserved with regards to the number and location of the clusters. However, in addition to the constant features of the axonal tracing patterns (see Figures 3, 4, 5, 6), we also found variations in these patterns across the investigated taxa. The number of cells within the clusters and also the size of the somata in each cluster vary. The position of the clusters in relation to each other and nervous structures seemed to be the most conserved and thus the most useful character to compare the cellular tracing patterns and to identify homologous clusters across different taxa. However, these features are not completely invariable, but variations are generally relatively minor. This variability might be caused by the fusion of ganglia and/or the strong variation of the morphology of the central nervous system. The lack of pedal clusters for *P. meckeli* could be more problematic. While our techniques appear to reliably stain central neurons projecting into the nerves, the long connectives between the cerebral and pedal ganglia in this species could exceed the distances over which nickel can travel in our assays.

### Correlation analyses

Under the assumption that the cerebral clusters are homologous we expected to find a correlation for the number of somata within the cerebral clusters between the investigated species. However, due to intra- and interspecific variability we expected the correlation to be low, as the homologisation was not based on the number only, and the absolute number of somata is still correlated to body size [11].

We found a very high correlation in somata number between *Aplysia* spp. and *Archidoris pseudoargus* and a very low correlation between *Aplysia* spp. and *Pleurobranchaea meckeli*. Furthermore, the overall correlation is highest within the N2 and N3 somata numbers and lowest in the N1 somata numbers. These differences might indicate a higher similarity in function within the CSOs which are provided by the N2 (tentacles and labial palps) and the N3 (rhinophores) than within the CSOs provided by the N1 (lips) and Nclc (bodywall). The higher correlation between *Aplysia* spp. and *A. pseudoargus* might also be caused by a more similar life history as *P. meckeli* is an active predator of mobile prey whereas *Aplysia* spp. feeds on algae and *A. pseudoargus* on sponges, which are both sessile organisms. However, we are very cautious with this hypothesis, which at current is highly speculative and needs more data to be tested.

### Homology hypotheses for the cerebral nerves

We postulate homology hypotheses for the cerebral nerves of the investigated species based on the observed conservation of their cellular innervation pattern of the respective nerves. The N1 innervates the lip region. The N2 has two branches (N2a, N2b) in all investigated opisthobranch taxa and is related to the anterior sensory organs. The two branches of the N2 innervate different cephalic areas, predicted to serve different functions [32]. In consequence we distinguish between four types of CSOs: lip – provided by the N1, ASOa – provided by the inner branch of the N2, ASOb – provided by the outer branch of the N2 and PSO – provided by the N3. The Nclc innervates structures of the head region which are involved in locomotion like the body wall or the cephalic shield nevertheless these structures could also perform sensory functions like mechano- or light reception [33-37].

Summing up, the conservative axonal tracing patterns of the cerebral nerves within the Opisthobranchia allow us to homologise these nerves. In consideration of earlier studies [10,11] our study supports the hypothesis [37] that the posterior Hancock’s organ in Cephalaspidea and the rhinophores in Nudipleura (Nudibranchia and Pleurobranchomorpha) and Aplysiomorpha are innervated by homologous nerves. The oral veil (Pleurobranchomorpha), lip organ (Cephalaspidea, Acteonoidea), and the labial tentacles (Aplysiomorpha and Nudibranchia) are also innervated by homologous nerves.

Nevertheless the axonal tracing method has its limitations. The first limitation is the size of the species: the techniques employed here require not only that the CNS be dissected without damage but that the individual nerves be large enough to manipulate (personal note: our limits have been species with a minimum body size of 0.5 cm). The second limitation is the number of specimens required, 5 to 10 undamaged replicates are needed for each nerve. Further restrictions have been discussed in previous studies [10,11]. A third limitation is that backfilling using NiLys does not permit evaluation of whether some cells might have axons in more than one nerve, thus complicating our analysis. Future work could better employ fluorescent labels which might allow double labelling resulting from backfilling of more than one nerve at a time.

### Primary homology hypotheses for the CSOs

As described above we divided the CSOs into the lip and the categories, ASOa and ASOb and PSO (Table 2), innervated by three homologous nerves (N1-N3).

Based on the hypotheses of homology for the cerebral nerves we postulate preliminary homology of the lip, which is innervated by the N1. Morphological and immunocytochemical investigations of the CSOs [38-43] indicated that the lip is supposed to be primarily a contact chemoreceptor organ. It can be assumed that the lip has the same function in all gastropods and represents a homologous structure throughout this taxon.

We also postulate homology of the various ASOs innervated by the N2, including labial tentacles, oral veils, oral tentacles, lip organ and the anterior part of the Hancock’s organ as it was defined by Edlinger [7]. The immunocytochemical and ultrastructural investigations [39,41-43] indicated that the ASOs are primarily mechano- and contact chemoreceptor organs. As discussed earlier we distinguish between two types/parts of the ASOs provided by the two different branches of the N2. Although homologies of types ASOa on the one hand and ASOb on the other hand are most likely, axonal tracing of the single branches of the N2 is necessary to homologize these parts of the nerve and in consequence to propose a homoloy hypothesis for the respective CSOs, as in some opistobranch species the anatomical differentiation between the ASOa and ASOb is clear but in other species it is not. In the current study due to the lack of specimen we only traced the entire nerve.

The third postulated homology is between the PSOs which are innervated by the N3 and supposed to be primarily olfactory [43,44]. In our opinion, the posterior Hancock’s organ of Cephalaspidea, as described in Edlinger [7] and the rhinophores (both innervated by the N3) are homologous structures throughout the Opisthobranchia, as has been stated earlier [7,8,17]. Our data confirmes this homology hypothesis as we found similar axonal tracing patterns for the cerebral nerve (see N3 in our definition) which innervates the rhinophores of Nudipleura (Nudibranchia and Pleurobranchomorpha) and Aplysiomorpha, and the posterior Hancock’s organ of the Cephalaspidea [11]. Furthermore similar neurotransmitter contents and similar sensory functions of the rhinophores and the posterior Hancock’s organ have been discussed [43]. Therefore the primary homology hypotheses proposed here are not only supported by the axonal tracing patterns, but also by the specific functions of the CSOs deduced from previous immunocytochemical data.

In the case of the Sacoglossa, where the rhinophores are innervated both by the N2 and N3 [8, pers. obs.] a homology to other rhinophores is questionable and needs further investigation.

The cerebral nerve Nclc does not seem to correspond to a primary sensory organ.

## Conclusion

In conclusion, we propose the usage of axonal tracing patterns as a new approach for the homologisation of nerves between relatively closely related species. The axonal tracing technique reveals a morphological character complex which serves to identify neuronal structures and consequently to homologise cerebral clusters and therefore serves as an additional character complex to homologise cerebral nerves. This study also confirms investigations [21,45] which postulated, that internal nervous structures are highly conserved during evolution. Due to their complexity and the known conservation of many neuronal characteristics [23,24,26,27] we believe the reconstructed innervation patterns to have a higher complexity than ganglionic structures. Therefore we propose that cellular innervation patterns are more suitable to distinguish between homologous and analogous nerves. Moreover, this neurobiological character complex may be usable for other investigations on similar organ systems, not only within the Opisthobranchia or Gastropoda. Furthermore it may be possible that the cellular innervation patterns contain a phylogenetic signal on a larger taxonomic scale.

## Methods

### Material

Four species of opisthobranch Gastropoda were investigated within this study, *Aplysia californica*Cooper, 1863 and *Aplysia punctata*Cuvier, 1803 (Aplysiomorpha), *Pleurobranchaea meckeli*Leue, 1813 (Pleurobranchomorpha) and *Archidoris pseudoargus* (Rapp, 1827) (Nudibranchia). The size variability of tracing patterns was taken into consideration by using specimens of similar size for all replicates.

*Aplysia punctata* (15 – 20 cm body length) and *Archidoris pseudoargus* (7 – 10 cm body length) were collected in the wild at Roscoff (Brittany, France). The animals were cultured in our lab in Frankfurt, maintained in closed seawater aquaria at 17°C, under ambient light and fed with frozen and defrosted pieces of the green algae *Ulva lactuca* and *Polysiphonia* spp. which were collected in the same location*.* Live *Aplysia californica* was purchased from the Aplysia Resource Facility of the Rosenstiel School of Marine and Atmospheric Sciences at the University of Miami (Florida, USA), and shipped and maintained in our lab in Frankfurt under earlier mentioned conditions and also fed with defrosted *U. lactuca* and *Polysiphonia* spp.. *Pleurobranchaea meckeli* (9 – 10 cm body length) was collected in Blanes (Spain) by fishermen via dredging at depths of 60 to 80 meters. In this case, preparations and axonal tracing experiments were performed immediately after collection of the animals.

### Neuroanatomy

Specimens were relaxed with an injection of 7% MgCl_2_ and fixed in 6% formaldehyde in seawater or with 4% paraformaldehyde in phosphate buffered saline, for at least 24 hours. For dissection the fixative was replaced with 70% ethanol.

### Cellular innervation patterns/axonal tracing

Animals were relaxed with an injection of 7% MgCl_2_ and the central nervous system, consisting of the cerebral, pleural, and pedal ganglia, was removed and placed in a small Petri dish containing filtered artificial seawater (ASW, Tropic Marin, REBIE, Bielefeld, Germany) as saline. We followed the procedures of Croll and Baker [46] for Ni^2+^-lysine (Ni-Lys) tracing of axons. The major cerebral nerves of four species (Table 1) were traced with approximately ten replicates for each nerve per species. Therefore the nerves of the right cerebral ganglion were dissected free from the connective tissue. In addition replicates for the cerebral nerves of the left hemisphere were performed in order to check for bilateral symmetry of tracing patterns. The nerves were cut and the distal tip was gently drawn into the end of a tightly fitting glass micropipette using suction provided by a 2.5 ml syringe attached with polyethylene tubing. The saline in the micropipette was replaced by a Ni-Lys solution (1.9 g NiCl-6H_2_O, 3.5 g L-Lysine freebase in 20 ml double distilled H_2_O) and the preparation was incubated for 12–24 h at 8°C to allow transport of the tracer. The micropipette was then removed and the ganglia were washed three times in ASW. The Ni-Lys was precipitated by the addition of five to ten drops of a saturated rubeanic acid (Dithiooxamide) solution (Sigma-Aldrich) in absolute dimethylsulfoxide (DMSO). After 45 minutes the ganglia were transferred to 4% paraformaldehyde and fixed for 4–12 h at 4°C. Thereafter the ganglia were dehydrated by an increasing ethanol series (70/80/90/100/100% each 10 minutes), cleared in methylsalicylate and mounted dorsal side up in Entellan (VWR International) on a glass slide. Our criterion for adequate staining was a uniformly dark blue nerve as it joined the ganglion. This is an indication for intact axons [22,47]. The Ni-Lys tracings were analysed by light microscopy (Leica TCS 4D). Camera lucida drawings were digitalised following the method of Coleman [48] adapted for CorelDRAW X4.

### Correlation analyses

For the correlation analyses we used the Pearson Correlation Free Statistics Software provided by Wessa (Free Statistics Software, Office for Research Development and Education, version 1.1.23-r6, URL http://www.wessa.net/), testing the numbers of somata within the cerebral clusters for the investigated species.

## Competing interests

The authors declare that they have no competing interests.

## Authors’ contributions

AKK and RPC designed the concept of the study, provided helpful discussions during data analysis and revised the manuscript; SES designed the experiment, sampled the specimens, performed the analyses and wrote a first draft of the manuscript. All authors read and approved the final manuscript.

## Authors’ information

AKK is associate professor for phylogeny and systematics. Her expertise lies in phylogeny and evolution of Heterobranchia (Mollusca, Gastropoda). RPC is a professor of physiology and biophysics. His expertise lies in molluscan neurobiology and the evolution of the nervous system. SES did his PhD studies on the evolution of cephalic sensory organs in the group of AKK. This contribution is part of these studies.
